# Supplementary material for: Dietary Problems Are Associated with Frailty Status in Older People with Fewer Teeth in Japan
Source: Int J Environ Res Public Health. 2022 Dec 5;19(23):16260. doi: 10.3390/ijerph192316260 (PMC9738370; doi:10.3390/ijerph192316260)
Supplement: Supplementary file 1 [file ijerph-19-16260-s001.zip › ijerph-2028396-supplementary.pdf]

Supplement Table S1. Characteristics of non-frailty and frailty participants

	All participants n = 160	Non-frailty n = 117	Frailty n = 43	P-value[a]
Age (median (25%–75%))	82 (79–86)	81 (78–86)	84 (80–87)	<0.019[c]
Female	72.5%	70.1%	79.1%	0.259[b]
Education				
≤ 9 years	50.0%	47.0%	58.1%	
10-12 years	33.1%	35.0%	27.9%	0.376[b]
≥ 13 years	16.9%	17.9%	14.0%	
Single/never married	54.4%	51.3%	62.8%	0.195[b]
Living alone	29.4%	29.9%	27.9%	0.804[b]
Care situation				
Independent	66.2%	75.2%	41.9%	
Requiring assistance	13.8%	7.7%	30.2%	<0.001[b]
Requiring long-term care	20.0%	17.1%	27.9%	
No ability to fill out forms by oneself	39.4%	30.8%	62.8%	<0.001[b]
Smoking				
Never	80.0%	80.3%	79.1%	
Past	15.0%	13.7%	18.6%	0.245[b]
Current	5.0%	6.0%	2.3%	
High alcohol consumption (≥ 3 cups of drinks per day)	5.0%	5.1%	4.7%	0.902[b]
Cognitive decline [d]	18.4%	16.8%	30.0%	0.051[b]
Medicine (≥ 3 per day)	74.4%	67.5%	93.0%	0.001[b]
CCI (median (25%–75%))	0 (0–1)	0 (0–1)	1 (0–1)	<0.001[c]

[a] Difference between the non-frailty and frailty group

[b] P for the chi-square test

[c] P for the Wilcoxon signed-rank test

[d] Excluding participants with missing value (n =8)

CCI, Charlson comorbidity index

Supplement Table S2. Distributions of SOF index between the ≥20 teeth and <20 teeth group

	All participants n = 160	≥20 teeth n = 63	<20 teeth n = 97	P-value[a]
SOF index (Number of items)				
0	38.1%	30.2%	18.6%	
1	82.5%	54.0%	47.4%	0.057[b]
2	38.1%	14.3%	28.9%	
3	6.2%	1.6%	5.2%	

[a] Difference between the ≥20 teeth and <20 teeth group

[b] P for chi-square test

Supplement Table S3. Proportions of denture use between the ≥20 teeth and <20 teeth group

	All participants n = 160	≥20 teeth n = 63	<20 teeth n = 97	P-value[a]
Denture use				
Upper denture use	59.4%	17.5%	86.6%	<0.001[b]
Lower denture use	58.1%	25.4%	77.3%	<0.001[b]

[a] Difference between the ≥20 teeth and <20 teeth group

[b] P for chi-square test
